# Improved cortical boundary registration for locally distorted fMRI scans

**DOI:** 10.1371/journal.pone.0223440

**Published:** 2019-11-18

**Authors:** Tim van Mourik, Peter J. Koopmans, David G. Norris

**Affiliations:** 1 Radboud University Nijmegen, Donders Institute for Brain, Cognition and Behaviour, Nijmegen, The Netherlands; 2 Erwin L. Hahn Institute for Magnetic Resonance Imaging, Essen, Germany; Center for Neuroscience and Regenerative Medicine, UNITED STATES

## Abstract

With continuing advances in MRI techniques and the emergence of higher static field strengths, submillimetre spatial resolution is now possible in human functional imaging experiments. This has opened up the way for more specific types of analysis, for example investigation of the cortical layers of the brain. With this increased specificity, it is important to correct for the geometrical distortions that are inherent to echo planar imaging (EPI). Inconveniently, higher field strength also increases these distortions. The resulting displacements can easily amount to several millimetres and as such pose a serious problem for laminar analysis. We here present a method, Recursive Boundary Registration (RBR), that corrects distortions between an anatomical and an EPI volume. By recursively applying Boundary Based Registration (BBR) on progressively smaller subregions of the brain we generate an accurate whole-brain registration, based on the grey-white matter contrast. Explicit care is taken that the deformation does not break the topology of the cortical surface, which is an important requirement for several of the most common subsequent steps in laminar analysis. We show that RBR obtains submillimetre accuracy with respect to a manually distorted gold standard, and apply it to a set of human in vivo scans to show a clear increase in spacial specificity. RBR further automates the process of non-linear distortion correction. This is an important step towards routine human laminar fMRI for large field of view acquisitions. We provide the code for the RBR algorithm, as well as a variety of functions to better investigate registration performance in a public GitHub repository, https://github.com/TimVanMourik/OpenFmriAnalysis, under the GPL 3.0 license.

## Introduction

Investigation of the BOLD response with functional MRI at the level of the cortical layers has become increasingly popular over the the last decade [1, 2]. Activation levels differ at the laminar scale [3] and they can vary depending on the performed task [4, 5]. Laminar signals have the potential to reveal information about the underlying neuronal processes within a cortical region, as the signal from different layers may be associated with feed forward or feedback signals [6, 7]. However, layer specific analysis comes with great methodological challenges.

The thickness of the cerebral cortex varies between 1 and 4.5 millimetres [8, 9]. Identifying individual layers therefore ideally requires sub-millimetre resolution, at the cost of signal to noise ratio (SNR) per voxel. On top of this, a functional experiment ideally requires a Repetition Time (TR) on the order of several seconds. Layer specific investigations are hence best conducted at higher field (≥7 Tesla) for improved SNR, but high field strength also have some disadvantages [10]. The inhomogeneities of the static magnetic field *B*_0_ can cause non-linear distortions when a fast acquisition scheme like Echo Planar Imaging (EPI) is used [11]. Distortions primarily present themselves in the phase-encoding direction as a function of the bandwidth per pixel and static field strength [12]. As layer specific analysis requires high spatial precision, non-linear distortions are particularly problematic. $T_{2}^{*}$-weighted images usually have insufficient contrast to segment the cortical grey matter, so instead one needs to identify the cortical boundaries from a different scan. This is typically a high-contrast *T*_1_-weighted anatomical scan that can be segmented with tools such as FreeSurfer [13], CBS Tools [14], or BrainVoyager [15]. However, as the anatomical scan is undistorted, it may not sufficiently overlap with the functional scan.

Several potential solutions have been proposed for providing accurate alignment of functional images with an anatomical image. Early papers circumvent the problem by segmenting only a small piece of straight cortex [16, 17]. It is also possible to resort to different acquisition schemes like FLASH, which do not suffer significant distortions, but at a heavy cost in temporal resolution. Koopmans et al. combine this with a vertex based realignment procedure based on the Stripe of Gennari [3]. This approach, however, is highly specific to parts of the primary visual cortex that show a myelinated band in the middle of the cortex (Stripe of Gennari), and does not generalise to the rest of the brain. Additionally, when the acquisition only covers a small part of the brain, cortical reconstruction algorithms may easily fail, as they are often based on whole-brain templates. Yet another alternative is to acquire an anatomical image with the same EPI readout and field of view as the functional image, such that the two volumes are similarly distorted [18]. However, this assumes that field inhomogeneities, and with it the distortions, do not change between acquisitions. Solutions in which T1-contrast is computed from the functional acquisition directly do not have this problem, for example the VASO sequence [19], or 3D-EPI with a variable TR [20]. Classically, *B*_0_ maps have been used for correcting distortions [21], yet this is typically used on low resolution images [22] and commonly accepted thereby not to be precise enough for sub-millimetre precision. Promising advances have also been made for high-resolution dynamic correction [23], but to date these have not been tested at sub-millimetre resolution. Additionally, for diffusion MRI a combined registration-segmentation method (regseg) has been developed that may be portable to high resolution laminar fMRI. [24].

Ideally, there would be an accurate cross-contrast (*T*_1_ to $T_{2}^{*}$) registration algorithm, but this is a notoriously hard problem. The combination of the warping of images and the unknown relation between contrasts creates a vast parameter space that is difficult to solve in a coregistration procedure. While algorithms like AFNI’s 3dQWarp [25] technically support cross-modal cost functions, the documentation acknowledges that such usage is rather experimental and in our experience indeed does not reach submillimetre accuracy. In general, no algorithm currently exists that verifiably corrects non-linear distortions in high resolution low-contrast images up to the laminar specific level, and works either on partial or whole-brain images.

One way to search through relevant information in the image domain is to try and detect the grey-white matter boundary and match this between volumes. This is using *geometric* information on top of *volumetric* information and forms the basis of Boundary Based Registration (BBR) [26]. A three-dimensional cortical reconstruction of the grey-white matter boundary is created on a high contrast anatomical image and serves as a basis for the coregistration. While a functional image is too low in contrast for generating a cortical reconstruction, it can be used in the registration procedure. The contrast is sufficient to optimise the average contrast across the boundary in order to achieve a better realignment. This was proposed for linear registrations and proven to be an exceedingly robust method. We here extend BBR to work recursively and effectively produce a cross-contrast non-linear registration. Our aim is to provide accurate within-subject submillimetre registration for whole-brain or partial brain images for standard high-resolution EPI sequences to make laminar fMRI analysis more accessible to a wider audience. The algorithms can be performed on the the functional data, without additional acquisition of additional scans, and without reinterpolation as a result of a non-linear warping. Importantly, the procedure produces a smooth deformation field that does not alter the topological properties of the mesh, such that the resulting surfaces can naturally be used in subsequent automatic layering and further layer specific analyses [27, 28].

## Methods

Boundary Based Registration (BBR) is based on a cortical construction of the boundary between the white matter and grey matter, and the grey matter and the CSF (pial surface) [26]. This surface is generated on an anatomical scan, as the contrast of the functional scan may not be good enough for segmentation. In order to register the two volumes, the constructed surface is moved to the functional image. The average contrast across the grey-white matter boundary is computed and used as a cost function to optimise the transformation parameters of the registration. Versions of BBR are implemented in FreeSurfer (bbregister) [13] and FSL (flirt -cost bbr) [29]. This method is powerful enough to be able to register the volumes, based on only a small part of the brain [26], suggesting that local application can also be successfully employed. Especially for high resolution (laminar) fMRI, the anatomical and functional volumes contain detailed information about the gyrification that can be used for registration. We hence propose to extend BBR with a hierarchical strategy [30]. By recursively applying BBR at diminishing scales as a series of linear transformation, the volumes are effectively non-linearly registered.

The way in which the mesh is divided is based on a three-dimensional cuboid lattice consisting of the set of neigbourhoods $N^{d}$ at various depths *d*, decreasing in size. At each depth, $N^{d}$ is divided into eight equally sized cuboids (two in each dimension) $N^{d+1}$ until a user specified threshold size is reached. All vertices that make up the brain mesh are divided over the elements of $V^{d}$, the set of vertices within $N^{d}$. The number of vertices in each neighbourhood may well vary between regions.

Registration is performed iteratively, from the largest scale (*d* = 0) to the smallest (*d* = *d*_*max*_). For each element $N_{i}^{d}$, a registration is computed by means of a boundary based registration algorithm [26] applied to $V_{i}^{d}$. In short, this is an edge detection algorithm that maximises the average contrast across the white matter surface. Contrast is defined as the (optionally weighted) gradient of samples on either side of vertices within the mesh. For the optimisation procedure we use MATLAB’s gradient descent method fminsearch (default parameters) to optimise registration parameters as a function of contrast. The same sampling method and cost function were used as proposed by Greve & Fischl [26].

Applying the computed transformations directly to $V^{d}$ could easily ‘break the mesh’ at the edges of neighbouring regions as their continuity is not guaranteed. This is a serious problem, as subsequent steps in laminar analysis (like the level set methods [31]) require the mesh to be a topological sphere: a non-intersection closed surface without holes. In order to ensure continuity we use a control point based strategy [30]: let the edges and corners of the neighbourhoods $N^{d}$ define a deformable lattice. For each computed transformation in a neighbourhood, a resulting displacement vector is assigned to all of its corner points. After all transformations at a depth level are computed, the median is taken of all displacement vectors for each control point, thus representing a resultant vector based on adjacent neighbourhoods. In order to further increase robustness (but at the cost of specificity), the displacement vectors may subsequently be adjusted based on their direct neighbours:

$$\begin{array}{c} \vec{d}=\alpha \vec{d}+(1-\alpha )M\left(\vec{x}\right), \end{array}$$ (1)

where $M(\vec{x})$ denotes the mean displacement of the neighbourhood. The balance between local matching and global smoothness is given by the *α* parameter and Collins et al. [30] experimentally found *α* = 0.5 yields an acceptable balance. We recommend higher values for *α*, as our primary goal in laminar analysis is the local matching. We suggest additional ways of increasing robustness in the next section.

As all control points within $N^{d}$ now have displacement vectors associated with them, $V^{d}$ can be displaced proportionally to the distance to their closest control points. A way of describing this is by defining transformation matrix **T**, such that it satisfies:

$$\begin{array}{c} D_{i}^{d}=TV_{i}^{d}, \end{array}$$ (2)

where the set of displacement vectors is denoted by **D**^*d*^, for which each *i*’th element contains 8 vectors, one for each corner of the cube. This is a typical linear regression equation and could hence trivially be solved for **T**. However, a least squares solution is required as the system is overdetermined with eight corner points and only a [4*X*4] transformation matrix. This necessarily requires an approximation of the deformation vectors that may result in discontinuities with respect to adjacent neighbourhoods. A preferred method is to divide the cube into six tetrahedra by means of Delaunay triangulation [32] and compute a separate matrix for each of them. For each tetrahedron, Eq 2 represents a determined system, for which the solution for adjacent tetrahedra is guaranteed to be continuous. Effectively, this division increases the degrees of freedom of the deformation field to satisfy our requirement of continuity. Having found the transformations, they can readily be applied to adjust the position of $V^{d}$.

This procedure is repeated for all depths levels *d*. Whenever the number of vertices in $N^{d}$ is smaller than a user specified minimum, the transformation for that region is set to the identity matrix.

### Robustness

**Fig 1:**
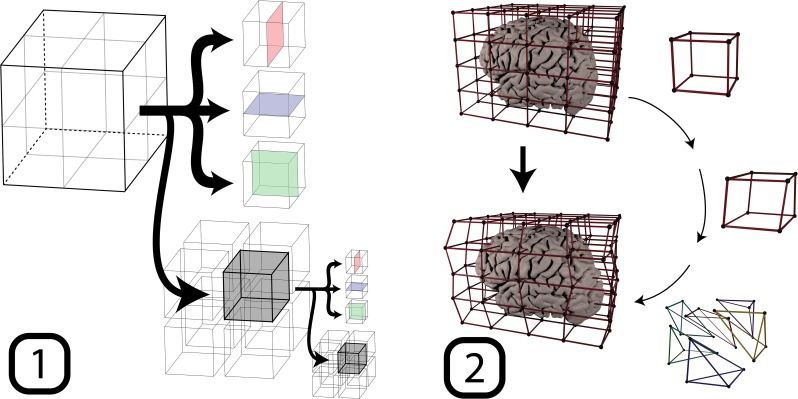
A schematic of the workflow. First, the volume is recursively broken up into parts, for which registrations are computed by means of the edge detecting boundary based registration method. Based on the transformations found in this step, the second step is the updating of a cuboid lattice. The control points within the lattice are updated and applied to the boundaries. Specifically, this was performed based on a tetrahedral division of the cube. For each tetrahedron, a displacement field was computed and applied to the vertices within the tetrahedron.

With the exponential increase in number of neighbourhoods as a function of depth level, the number of registrations easily reaches into the hundreds. The high number of Degrees of Freedom (DoF) of the algorithm therefore inescapably increases the probability of misregistrations. The algorithm ensures robustness and continuity in several ways, by computing displacement vectors as a weighted average of all surrounding neighbourhoods, and by an (optional) additional smoothing of displacement vectors based on adjacent control points, as mentioned above. Additionally, we compute a transformation within a neighbourhood not only for the entire neighbourhood, but also for six subregions. By splitting $V^{d}$ separately into two in the *x*, *y*, and *z* dimensions, six cuboids are created for which the registration is repeated. This procedure is illustrated in Fig 1. The resulting displacement vectors are added to the respective list for each control point.

### Parameters

The algorithm can look for any combination of translation, rotation and scaling in the *x*, *y*, and *z* dimensions. It will divide the volume until it reaches a user defined minimum size or number of vertices, and a transformation will be computed. We here focus on registration in the phase encoding direction only, i.e. translation and scaling in the *y*-direction, as this is the most common type of distortion when an Echo Planar Imaging sequence is used [11]. We set the minimum size of a neighbourhood to 4 voxels and the minimum number of vertices to 100. The smoothing factor with respect to adjacent vertices in the lattice from Eq 1 was set to *α* = 0.9. Experimentally, we have found that this still yields robust results while still being highly weighted towards specificity. In contrast, Collins et al. [30] describe an *α*-level of *α* = 0.5, which is likely to do with the fact that their purpose of template matching and segmentation prioritises robustness over specificity. While the original implementation of BBR recommends using subsets of vertices for parts of the algorithm [26], we here use all vertices at all stages. This may be redundant for registrations at the large scale, but as it is a small addition in computation time, and because errors in early iterations may propagate further downwards, we chose to incorporate all vertices. Finding the best selection of parameters may still require some iterations for a given data set. It is, however, a substantial improvement with respect to the arduous job of manually matching small patches of cortex within a very limited field of view.

### Validation

Assessing the quality of the registration performance proved challenging. While there is a clear theoretical relationship between the distortion size and the parameters of the acquisition [21], in practice it is difficult to find a gold standard for the submillimetre accuracy that we are aspiring to. Tools to convert field maps to estimates of voxel displacement are usually heavily smoothed and cannot account for subject movement in the scanner or field changes between acquisitions. We hence created our own gold standard by acquiring an undistorted FLASH image and manually distorting it based on a field map. This way, the exact distortions were known in order to test if we could find them back with RBR.

A high resolution whole brain multi-echo FLASH image [33] was acquired at a 3T Siemens Scanner, TR = 95 ms, *α* = 20°, bandwidth = 170 Hz/px, [0.75 mm]^3^. GRAPPA was used for three-fold in-plane acceleration. The echo times ranged from 5.88 ms to 78.96 ms with an echo spacing of 8.12 ms. The average of the last seven echoes was used, as the first three contained little contrast. This was accompanied by a whole brain MPRAGE acquisition that was used for the FreeSurfer cortical reconstruction v5.1, default parameters, TR/TE/TI/*α* = 2300 ms/3.15 ms/1100 ms/8°, [0.8 mm]^3^. A field map was acquired to realistically distort the FLASH image. The resolution was [3.5 mm X 3.5 mm X 2.0 mm], TR/TE1/TE2/*α* = 1020 ms/10 ms/12.46 ms/90°, bandwidth = 260 Hz/Px.

The cortical reconstruction from FreeSurfer’s recon-all [13] was coregistered to the FLASH image using a 6 DoF linear registration with a custom MATLAB implementation of BBR. In order to distort the cortical surface, a voxel displacement map (VDM) was computed using SPM field map tools [34]. In order to taper unrealistically large displacements, and to move non-displaced vertices away from zero, the VDM was first transformed by a cubic root, after which it was applied in the anterior-posterior direction to the boundaries. Vertices were on average displaced by 2.56 mm (3.4 voxels), which is considerably more than could be tolerated for a laminar specific experiment. The distribution of displacement values was bimodally distributed away from zero with the specific goal to let RBR find the displacement and yield a sharp unimodal distribution around zero, as close to a delta distribution as possible.

Additionally, 12 brain scans were obtained from a 7T scanner to test RBR. One was excluded at an early stage of the analysis because of poor quality of the cortical reconstruction, despite manual efforts. We used 3D EPI [35], [0.93 mm]^3^, TR/TE/*α* = 2768 ms/20 ms/14°, bandwidth = 1167 Hz/pixel, phase encoding direction: A -> P, matrix size = 204^2^, effective echo spacing = 0.25 ms. The boundaries were created by FreeSurfer on a whole-brain MP2RAGE (1.03 mm^3^, TR/TE/TI1/TI2 = 5000 ms/1.89 ms/900 ms/3200 ms) [36]. BBR was performed by bbregister with a full affine transformation (12 DoFs) on the mean of the functional images and subsequently, the boundaries were imported to MATLAB. Overlaying the registered boundaries on top of the EPI image showed clear local geometrical distortions in the phase encoding direction, related to field inhomogeneity [21]. These distortions were of the order of several millimetres within a single volume. We corrected this with RBR and investigated its performance.

It must be noted that it is challenging to find an objective metric by which the quality of the registration could be quantified. The true distorted position is unknown and methods to approximate do not have the desired submillimetre specificity that is required. Moreover, if such metric existed, it could itself be used in the optimisation procedure. An alternative, however, is an investigation of RBR’s performance on the true volume compared to the performance on the volume with different levels of added noise. Assuming that the algorithm has the best performance on the original data, the displacement with respect to the no-noise condition is expected to increase when more noise is added. Note that this does not make a statement about the correctness of the result. To investigate the RBR’s performance in the presence of noise, we added twelve different levels of white noise to the data, applied RBR and compared the displacement to the unsalted version. For this, we used the Average Absolute Distance (AAD) [26], defined to be the average distance that the cortical surface moves between the two sets of registrations.

#### Ethics statements

This study was approved by the DCCN CMO 2014/288 (Donders Centre for Cognitive Neuroimaging, Commissie Mensgebonden Onderzoek). CMO Arnhem-Nijmegen is an accredited IRB in the Netherlands. All participants provided written informed consent in accordance with its guidelines.

#### Participants

In total, twelve participants were recruited from the Donders Institute participant pool (7 male, mean age 23, range 18-26). One was excluded at an early stage of the analysis because of poor quality of the cortical reconstruction, despite manual efforts.

## Results

**Fig 2:**
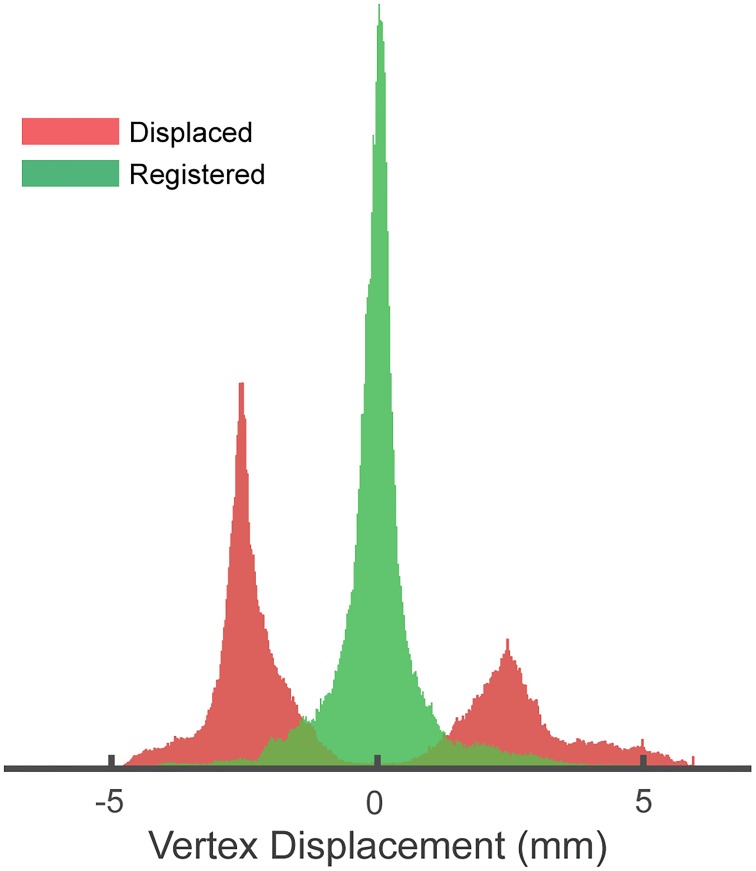
Histogram of the displacement of all 228,208 vertices within a single brain mesh. The red histogram shows the displacement after fieldmap based distortion with respect to a gold standard. The green shows the displacement after applying RBR with respect to the same standard. After registration, the histogram clearly shows a sharp zero-centered (no bias) distribution.

**Fig 3:**
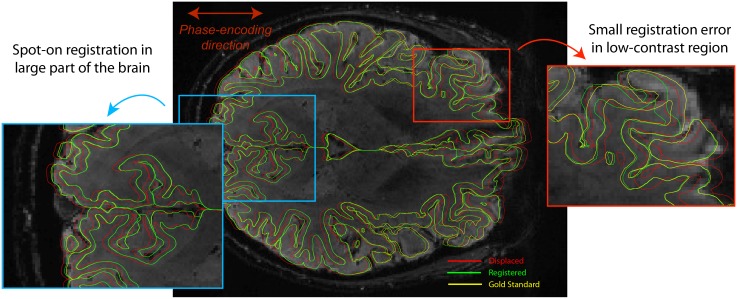
The middle slice of the registration performed on a manually distorted FLASH image (red boundaries). The registered surface (green) overlaps for the larger part of the brain almost perfectly with the gold standard (yellow). If the specificity is set to high values, there is a risk that errors start to appear in some low contrast regions. This largely depends on the balance between false positives and false negatives in terms of corrected regions.

We employed RBR on a constructed gold standard where the exact displacement was known. By taking an undistorted FLASH image and realistically distorting the cortical surface by means of a field map, we could test if we could retrieve the initial position of the boundaries. In Fig 2, we present a histogram of the displaced boundaries and the registered boundaries, both with respect to the true position. The registered boundaries clearly show a sharp distribution centred around the origin (*μ* = 0.027 mm). The FWHM of the distribution is 0.49 mm, showing that RBR provides accurate submillimetre registration. Additionally, Fig 3 shows a cross section (middle slice of the volume) of the registration. S2 Fig shows a colour coded figure with the difference in millimetres of the registrered boundaries with respect to the gold standard.

In most of the slice (and the volume), the registration accuracy is well within the submillimetre regime. However, especially in low contrast areas the algorithm may show some small inaccuracies. This mainly proliferates when there is also another gradient in the image (e.g. the pial surface) on which the algorithm starts to fix the boundaries. This is largely related to the fine line between obtaining sufficient specificity and overfitting the data.

**Fig 4:**
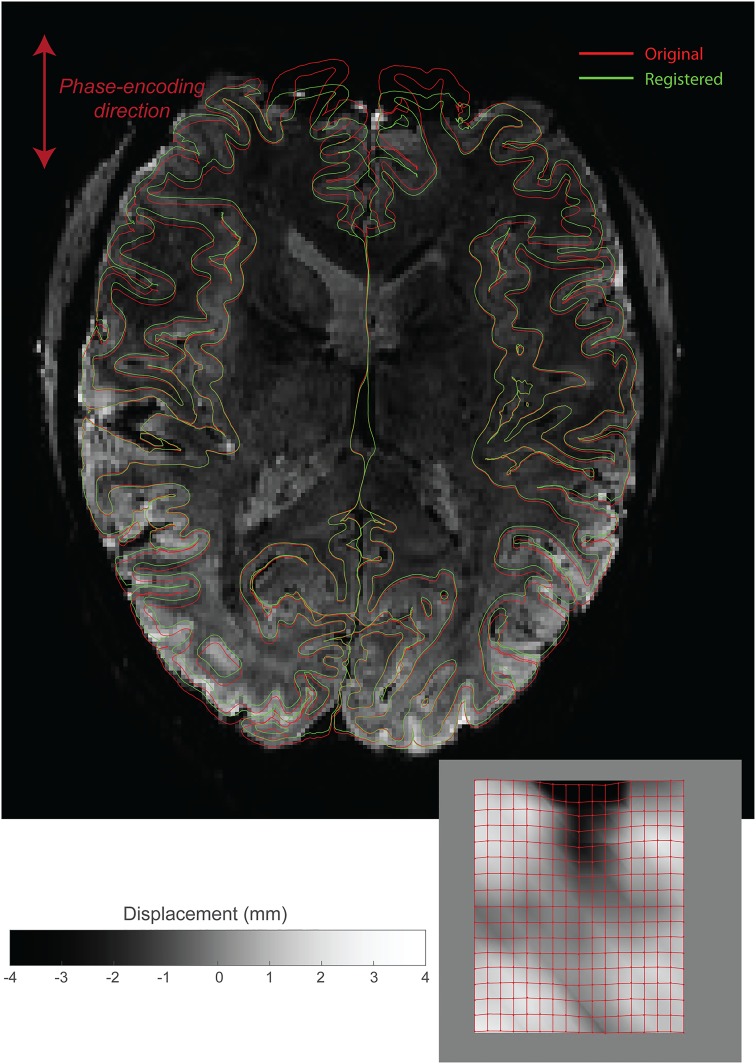
Distortion correction of 7T 3D EPI data, obtained with 0.93 mm isotropic resolution. In red, the original brain surfaces are shown after a 12 DoF registration performed by bbregister. The mesh in green is the updated mesh by means of the first stage RBR, 2 DoF (scale and translation in the PE direction). The green boundaries follow the white matter boundary much better. The voxel displacement map (lower right) shows displacements on the order of several millimetres. The control point lattice that was used to displace the boundaries are overlaid onto the displacement map. Similar images for all subjects can be found in the supplementary materials. For even better inspection, movie files for all subjects are included in the online supplemental materials.

We performed RBR on a (resting state) dataset intended for laminar analysis consisting of 11 subjects. The boundaries after a 12 DoF bbregister were recursively registered to the mean EPI images (0.93 mm isotropic resolution) and this yielded an updated cortical surface. The new surface followed the grey matter boundary in the volume visibly better than the unregistered one. In Fig 4 we present a single slice with both sets of boundaries overlaid on top of them, illustrating the improvement. We here present the data for a representative subject, and identical images for all other subjects are presented in S1 Fig.

By the nature of the algorithm the displacements are weighted averages of several iterations of correction. As a result, in regions of drop-out or low contrast and thereby high computational uncertainty, the algorithm will default to ‘no change’, especially in the finer iterations. However, in the earlier coarser iterations, low-signal regions may be drawn towards higher contrast regions in order to optimise spurious boundaries. As a result, highly distorted regions and drop-out regions may not be corrected accurately, of which the frontal region of Fig 4 is an example. It should be noted, however, that these regions are unlikely to be suitable for high-precision analysis and thereby outside the scope of the intended use cases of RBR.

**Fig 5:**
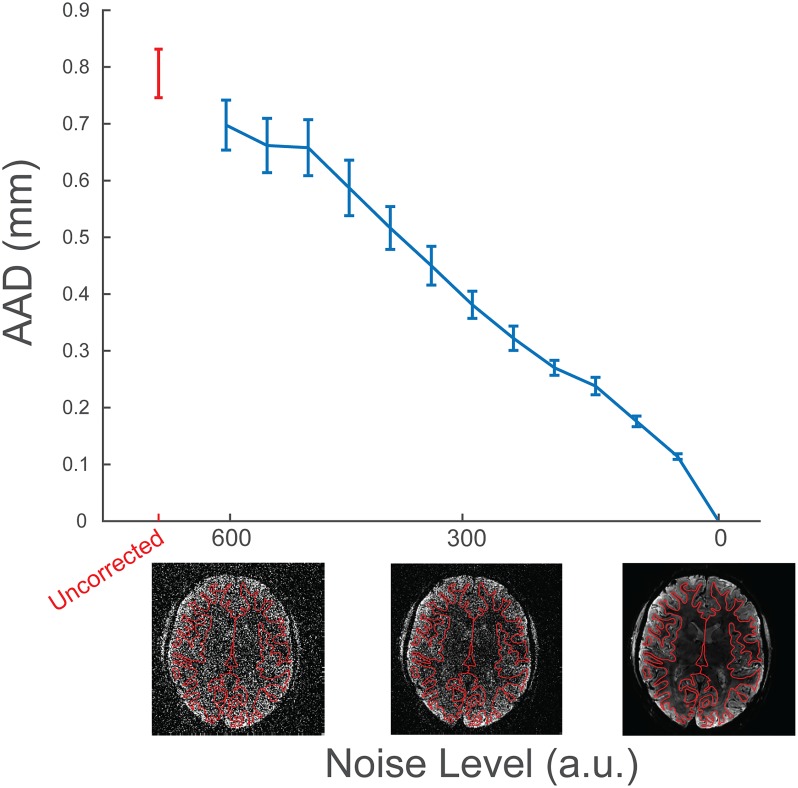
The effect of adding noise on the computed average absolute displacement with respect to a no-noise registration. In the absence of knowledge about the true distortions, the monotonic decrease of the AAD indicates that RBR converges to an optimum as a function of data quality.

Additionally, Fig 5 shows the AAD of the registration on salted images with respect to the registration on the no-noise volume. Even for highly noisy images, the registration improves somewhat with respect to the uncorrected boundaries. In the absence of a gold standard model of the distortions, the monotonic decrease of the AAD indicates that RBR converges to an optimum as a function of data quality.

### Data availability

All source code for the registration algorithm is freely available under the GPL 3.0 license at https://github.com/TimVanMourik/OpenFmriAnalysis.

The respective modules are also available in Porcupine and GiraffeTools, visual pipeline tools that automatically creates custom analysis scripts [37]. An example pipeline with all modules in the context of laminar analysis is set up in https://giraffe.tools/workflow/TimVanMourik/OpenFmriAnalysis.

All code to generate the images in this paper are available at the Donders Repository http://hdl.handle.net/11633/di.dccn.DSC_3015016.05_558. Specifically, the repository contains 1 subject for the FLASH acquisition and 11 experimental data sets for the MPRAGE-to-EPI distortion correction procedure (sequence parameters are described in the method section).

The data set associated with our distortion simulation on a FLASH image contains a single high-resolution high-SNR multi-echo FLASH volume and an anatomical scan with a FreeSurfer surface reconstruction. It provides all code to perform the field map based distortion simulations, the RBR non-linear distortion corrections of the simulated volumes, and the code to produce Figs 2 and 3.

The data set associated with the EPI distortion correction includes for each subject a structural scan with a FreeSurfer reconstruction and the mean of the functional acquisition. The raw time series are available upon request, but are not included as we do not perform any temporal analysis. We further include all derived files as produced by the MasterScript.m MATLAB script. This script first performs a linear boundary based registration and subsequently our proposed non-linear recursive boundary based registration. It further saves the registered cortical surfaces to a MATLAB readable format and outputs movie files that scroll through the brain with corrected and uncorrected surfaces as animated plots. The code proceeds with adding noise to the images (for the previously described AAD analysis), recomputes the non-linear registrations, and produces the base of Figs 4 and 5.

## Discussion and conclusions

In recent years, laminar specific fMRI analysis has come in reach for human in vivo experiments. Where standard fMRI analyses are done on a routine basis, this is hardly the case for layer specific analysis, not least because of the large amount of manual work that is involved. Aligning volumes to adjust for subject motion and non-linear distortion is one of the most challenging parts of the pipeline. With Recursive Boundary Registration (RBR) we here propose a solution to this problem by means of a recursive application of Boundary Based Registration. By using a control point lattice that forms the basis of deforming the mesh, the topology of the surface is preserved. This way, the mesh can easily be fed into next steps in making a cortical layering (Level Set Method) where it is an absolute necessity that the mesh is not broken as a result of the transformations. Due to the large number of degrees of freedom that are required to non-linearly deform a volume, we built in several methods to increase robustness. Nonetheless, as a mathematical certainty, increasing the sensitivity will increase the false positive rate, i.e. the risk of overfitting. It is up to the user to find the desired balance between them in the settings that we provide to control this balance. We hence provide the full code and data of all our experiments online, in order to aid users in adopting this method and fine-tuning the parameters for their use case. Guidance with respect to parameters, their defaults, and possible alternatives are documented in the code. Although RBR supports translation, rotation and scaling in any direction, it must be noted that our test cases were one-dimensional corrections. The dominant distortion in the phase encoding direction is typically an order of magnitude larger than in other directions and it is therefore logical to adjust the settings accordingly.

To ensure the functionality of this method, several conditions have to be met. A good reconstruction of the cortical surface is essential, as any inaccuracies will directly affect the contrast estimation and hence the registration optimisation. As the algorithm is based on edge detection, the volume to which it is registered needs to have sufficient contrast between white and grey matter. Furthermore, RBR is recommended to be initialised by a linear BBR registration. While RBR excels at subtle non-linear deformations, large displacement at any level may not be found in a rough multidimensional landscape through which the gradient descent method has to find its way to a minimum. In general, a gradient descent method may be difficult to monitor as it is susceptible to local minima trapping. A more robust algorithm may be a good improvement to RBR (e.g. using bounded gradient descents, or Monte Carlo sampling). Additional points of improvement could be the way in which the control point lattice is applied. While the subdivision into tetrahedra guarantees continuity across segments, it has a non-symmetrical orientation. The resulting displacement hence may show a residual bias as a result of the division. A smoother curve fitting technique to replace the current tetrehedral solution may be a valuable improvement, for example a diffeomorphic version as implemented in ANTS [38].

This algorithm in its current implementation focuses only on the *gradient* around the gray and the white matter, without looking at the intensity value. One could imagine this to be problematic when RBR is applied to a volume where a similar gradient of the same direction is present, for example the grey matter to CSF gradient. The algorithm may easily get ‘confused’ about which boundary it needs to converge on. In $T_{2}^{*}$-weighted images in functional studies, this is not usually a problem as the grey matter has a higher intensity value than both CSF and white matter. However, in standard anatomical scans with *T*_1_ contrast, this is not the case. As a result, RBR may fail due to the great similarity of contrast along both sides of the grey matter. An extension of RBR could focus on using more prior knowledge about the intensity (as opposed to merely the gradient), in order to inform the algorithm which boundary it should use. This may approach solutions like regseg [24] that was developed in the context of distortion correction for diffusion MRI and presents a combined registration-segmentation method that deals with excessively smooth gradients.

A common use of non-linear registration is template matching, for example in an anatomical-to-MNI normalisation. RBR has little potential for this type of usage as it is based on finding fine within-subject similarities in a volume, rather than the coarser type of between-subject similarities. More probable potential use cases may be could in the matching of subcortical brain structures that are described with a three-dimensional mesh. Similarly, there is a wide range of more deformable parts of the body (e.g. liver, heart, stomach, etc.) that might benefit from solutions like RBR. Additionally, RBR could find its use in developmental investigations of the brain. Pediatric longitudinal studies may cross-register surfaces obtained from older participants to earlier time points, as FreeSurfer cortical reconstructions get progressively less accurate for younger children.

We have shown that RBR automates the process of non-linear distortion correction in order to accommodate an extremely high specificity. This is a next step in automated processing of laminar analysis and more routine layer research. We have shown this yields submillimetre accuracy of registration. With the open source and modular way the code is written, the cost function, optimisation algorithm or deformation algorithm could easily be replaced with improved versions, to accommodate ongoing development. Finally, we concur with Saad [39] and Greve [26] in their conclusion to encourage visual inspection of the registration before reliance is placed on its accuracy.

## Supporting information

S1 Fig Distortion correction of 7T 3D EPI data, obtained with 0.93 mm isotropic resolution. In red, the original brain surfaces are shown after a 12 DoF registration performed by bbregister. The mesh in green is the updated mesh by means of the first stage RBR, 2 DoF (scale and translation in the PE direction). The green boundaries follow the white matter boundary much better. For better inspection, the online supplemental materials include movie files that scroll through the volume. The voxel displacement map (lower right) shows displacements on the order of several millimetres. The control point lattice that was used to displace the boundaries are overlaid onto the displacement map. These are single slice images of the registration performance for all other 10 subjects. More detailed information can be found in the online data sharing collection.
(EPS)

S2 Fig Registration performance of the FLASH data compared to a gold standard, as described in the Results section. The difference of the registered boundaries and the gold standard is colour coded in the figure. A detailed movie that displays the 3D information of the registration quality of the entire volume can be found in the online supplementary materials. The mid slice of the registration performed on a manually distorted FLASH image is shown. The image on the left shows the boundaries after applying a manual distortion. The image on the right shows the remaining distortion after RBR correction. The surfaces are colour coded with the displacement in millimetres with respect to the gold standard.
(EPS)
